# Mechanism of HIV protein induced modulation of mesenchymal stem cell osteogenic differentiation

**DOI:** 10.1186/1471-2474-9-33

**Published:** 2008-03-13

**Authors:** Eoin J Cotter, Herbert Shi Ming Ip, William G Powderly, Peter P Doran

**Affiliations:** 1 Clinical Research Center, School of Medicine and Medical Sciences, University College Dublin, Catherine Mcauley Centre, Nelson St., Dublin 7, Ireland; 2 School of medicine and Medical Sciences, University College Dublin, Belfield, Dublin 4, Ireland

## Abstract

**Background:**

A high incidence of decreased bone mineral density (BMD) has been associated with HIV infection. Normal skeletal homeostasis is controlled, at least in part, by the maturation and activity of mature osteoblasts. Previous studies by our group have demonstrated the ability of HIV proteins to perturb osteoblast function, and the degree of osteogenesis in differentiating mesenchymal stem cells (MSCs). This study attempts to further dissect the dynamics of this effect.

**Methods:**

MSCs were cultured under both osteogenic (cultured in commercially available differentiation media) and quiescent (cultured in basal medium) conditions. Both cell populations were exposed to HIV p55-gag and HIV rev (100 ng/ml). Time points were taken at 3, 6, 9, and 15 days for osteogenic conditions, while quiescent cells were treated for 1 week. Cell function (alkaline phosphatase [ALP] activity, calcium deposition, and lipid levels) and the activity of the key MSC transcription factors, RUNX-2 and PPARgamma were determined post-exposure. Also, in cells cultured in differentiating conditions, cellular levels of connective tissue growth factor (CTGF) were analysed using whole cell ELISA, while BMP-2 secretion was also examined.

**Results:**

In differentiating MSCs, exposure to HIV proteins caused significant changes in both the timing and magnitude of key osteogenic events and signals. Treatment with REV increased the overall rate of mineralization, and induced earlier increases in CTGF levels, RUNX-2 activity and BMP-2 secretion, than those observed in the normal course of differntiation. In contrast, p55-gag reduced the overall level of osteogenesis, and reduced BMP-2 secretion, RUNX-2 activity, CTGF levels and ALP activity at many of the timepoints examined. Finally, in cells cultured in basal conditions, treatment with HIV proteins did not in and of itself induce a significant degree of differentiation over the time period examined.

**Conclusion:**

These data demonstrate that the effect of HIV proteins on bone is dependent on the differentiation status of the cells that they are in contact with. The effect on bone cell signalling provides insights into the mechanism of HIV induced decreases in bone mineral density.

## Background

Osteoporosis is characterised by a severe loss of bone mass with consequent reduction in bone strength, leading to an increased fracture risk. Osteopenia is the lesser reduction of bone mass which precedes osteoporosis [1]. Recent studies have suggested an association between bone abnormailites, including osteoporosis, ostepenia, and osteolysis, and HIV infection [1,2]. However, the molecular and cellular mechanisms underpinning these HIV associated changes in bone biology remain unclear, with some, but by no means all, studies linking these abnormalities with the use of potent antiretroviral medications [3-11].

The balance between osetoclast mediated bone resorption and osteoblast mediated bone deposition is the essential feature of homeostatic bone remodelling. This process allows the regulation of skeletal growth and repair, and the maintenance of skeletal integrity. Osteoblasts (OB) and Osteoclasts (OC) are derived from different cell lineages, with OBs being formed by the differentiation of mesenchymal stem cell (MSC) precursors in the bone marrow, and OCs dervived from hematopoietic cells and are distantly related to other cell types such as monocytes and machrophages. Just as the bone producing and degrading functions of OBs and OCs balance each other, a complex arrangement of intercellular signalling between OBs and OCs allow their maturation, relative number, and activity to be regulated [9-13]. Vital to this intercellular signalling system are the bone morphogenic proteins, BMP2-9, which regulate osteoblast function and development; In the case of BMP2 and BMP7, an activity that is mediated via induction of the activation and expression of the transcription factor RUNX-2 [14-17].

Mesenchymal stem cells (MSC) are bone marrow derived pluripotent cells which differentiate into several mesenchymal cell types, including OBs and adipocytes (AC) [18]. Their differentiation is a tightly regulated and, as of yet, not fully elucidated process [19] However its is believed that the balance between transcription factors RUNX-2 (also called Cbfa1) and peroxisome proliferator activated receptor (PPARγ); plays a crucial role in controlling MSC development [19], with RUNX-2 driving a pro-osteogenic phenotype and PPARγ a pro-adipogenic one, Alterations in the ratio of OBs to ACs generated from MSCs have been clinically linked to decreased bone mass, with an increase in bone marrow AC content observed in both osteoporosis and osteopenia [19,20], while other conditions which lead to bone loss, such as treatment with glucocorticoids, have also been shown to increase the number of bone marrow ACs [19,21].

The widely accepted model of the progression of osteogenesis is that the differentiating mesenchymal stem cell proceeds through a number of functional stages, namely proliferation, matrix maturation, and mineralization. These stages are indicative of phenotypical transition of the pluripotent MSC to a committed osteoprogenitor, preosteoblast, early osteoblast and finally a mature osteoblast [22,23].

Molecules associated with induction of osteogenesis include connective tissue growth factor (CTGF), a 38-kDA protein expressed in a number of cell types including fibroblasts and MSCs. CTGF is a member of a family of cysteine rich proteins, other members of which are associated with a wide variety of biological processes such as embryonic development and repair [22]. In MSC differentiation, increased CTGF expression is associated with the early stages of osteogenesis (proliferation, matrix maturation), preceeding such other later osteogenic markers such as alkaline phosphatase activity and mineralization [22,23].

Previous studies by our group have demonstrated that treatment with the HIV proteins p55-gag and gp120 could impair osteoblast function, reduce secreted levels of BMP-2, osteocalcin and RANK-L, and reduce RUNX-2 expression and activity. In addition, our group has also previously published data showing that HIV-p55 and REV decrease and increase the degree of MSC osteogenesis respectively [24].

Herein we have extended our investigation into the effects of HIV proteins on MSC osteogenesis. Initially the effect of varying concentrations of an array of HIV1 proteins MSC osteogenesis was examined. Following that we examined the osteogenic markers of alkaline phosphatase activity and calcium deposition, as well as quantifying cellular levels of CTGF, secreted BMP-2 and RUNX-2 transcription factor activity over the time course of osteogenesis in the presence and absence of p55-gag and REV. Furthermore, to determine whether HIV proteins themselves can influence MSC differentiation, we treated non differentiating MSCs with REV and p55-gag and examined levels of calcium deposition, alkaline phosphatase activity, lipid levels as well as examining RUNX-2 and PPARγ transcription factor activity. The data presented herein demonstrates that although HIV p55-gag and REV do not in and of themselves induce MSC differentiation, they alter the timing and magnitude of important osteogenic signals and events throughout the natural history of MSC differentiation.

## Methods

### Cell Culture

A commercially available human Mesenchymal Stem Cell (derived from human bone marrow withdrawn from the posterior iliac crest of the pelvic bone of normal volunteers) line was obtained from LONZA, UK, and routinely cultured at 37°C, 5% CO_2 _in Mesenchymal Stem Cell growth media. Cells were differentiated by culturing in Osteoblast Differentiation media (LONZA UK) for 15 days. Cells were used between passage 3–6.

Cells were treated with varying concentrations of (10 ng/ml–100 ng/ml) HIV rev, p55-gag, TAT and gp120 proteins. Both *undifferentiated *and *differentiating *cells were treated with HIV proteins. REV and P55-gag have been previously shown by our group to affect osteogenesis over a 21 day time course at 100 ng/ml, a concentration previously determined to be representative of *in vivo *levels of HIV gp120 in untreated patients [11,24].

Viral proteins were obtained from the NIH aids reagent program [25]; *HIV-1 Rev (Wild Type;) *was obtained through the NIH AIDS Research and Reference Reagent Program, Division of AIDS, NIAID, NIH: HIV-1 Rev (Wild Type) from Dr. David Rekosh, Dr. Marie-Louise Hammarskjöld and Mr. Michael Orsini. *HIV-1*_*IIIB *_*p55 Gag; *was obtained through the NIH AIDS Research and ReferenceReagent Program, Division of AIDS, NIAID, NIH: HIV-1_IIIB _p55 Gag. *HIV-1 Tat Protein *The following reagent was obtained through the NIH AIDS Research and Reference Reagent Program, Division of AIDS, NIAID, NIH: HIV-1 Tat protein from Dr. John Brady and DAIDS, NIAID. *HIV-1 CN54 gp120*. The following reagent was obtained through the NIH AIDS Research and Reference Reagent Program, Division of AIDS, NIAID, NIH: HIV-1 CN54 gp120 from DAIDS, NIAID

### Alkaline phosphtase staining

Cells were cultured in a 96 well plate, and treated as described above. Cells were then washed in PBS and fixed with 100 μl ice cold 1:1 Ethanol:Acetone for 10 mins. After cell number determination (see below), cells were washed several times in PBS to remove any remaining stain, and allowed to air dry. SigmaFAST BCIP/NBT solution was prepared as per manufacturers instructions, and 100 ul of stain solution added to each well. The plate was then incubated for 30 mins at 37°C. Unstained, similarly treated wells, were also included as blanks. Following incubation, the remaining substrate solution was removed, the wells washed 3 times with 100 ul PBS, and allowed to air dry. The plate was then scanned at 300 dpi using a standard flatbed scanner.

The degree of staining was quantitated spectrophotmetrically; 100 ul 1%CPC was added to each well and the plates incubated with agitation for 30 mins (100 rpm). The plates were then read at 560 nm with a correction wavelength of 700 nm.

### Calcium Deposition

Cells were cultured in a 96 well plate, and treated as described above. Cells were then washed in PBS and fixed with 100 μl ice cold methanol for 10 mins. After cell number determination (see below), cells were washed several times in PBS to remove any remaining stain, and allowed to air dry. 100 ul of 40 mM alizarin red (pH 4) solution was added to each well. The plate was then incubated for 10 mins at room temperature. Unstained, similarly treated wells were also included as blanks. Following incubation, the remaining stain solution was removed, the wells washed 3 times with 100 ul PBS, and allowed to air dry. The plate was then scanned at 300 dpi using a standard flatbed scanner.

The degree of staining was quantitated spectrophotometrically; 100 ul 1%CPC was added to each well and the plates incubated with agitation for 30 mins (100 rpm). The plates were then read at 557 nm with a correction wavelength of 700 nm and alizarin red concentration determined using a standard curve of concentration vs. OD_557 nm_. Data was normalised to cell number per well.

### Lipid Staining

Lipid levels were assessed using a quantitative oil-red-o protocol adapted for use in a 96-well plate format. Briefly, undifferentiated MSCs were cultured and treated as described above. Cells were then washed in PBS and fixed with 100 μl formalin (8%) for 10 mins. After cell number determination (see below), cells were washed several times in PBS to remove any remaining stain, and allowed to air dry. A stock solution of Oil Red-O (sigma) was prepared (5 mg/ml 60% isopropanol). This was used to prepare a working solution 3.3 mg/ml, which was then filtered. 100 μl of this solution was added to each well, and left to stain for 15 mins. Post staining cells were washed (1 wash 60% isopropanol, 2 wash PBS), and allowed to air dry before being de-stained with 80 μl 1% CPC (10 mins, agitation [100 rpm]). Again unstained wells containing similarly treated cells were included as blanks for each treatment. Each plate was read at 530 nm, with a reference wavelength of 700 nm, and Oil Red-o concentration determined using a standard curve of concentration vs. OD_530_. Data was normalised to cell number per well. In undifferentiated MSCs, levels of lipid staining were modest, the staining being visible to the naked eye, but differences being undeterminable.

### Cell Number Determination

After fixation cells were washed twice with PBS and allowed to air dry, before being stained with ponceau red (100 μl per well, 10 mins). Unstained wells containing similarly treated cells were included as blanks for each treatment. The cells were then washed twice with 200 μl PBS per well to remove excess stain, air dried and destained (100 μl PBS per well, 10 mins, agitation [100 rpm]). The plate was then read at 540 nm, with a reference wavelength of 700 nm, and cell number per well determined using a standard curve of cell no vs. OD_540_. Unstained wells containing similarly treated cells were included as blanks for each treatment.

**Figure 1:**
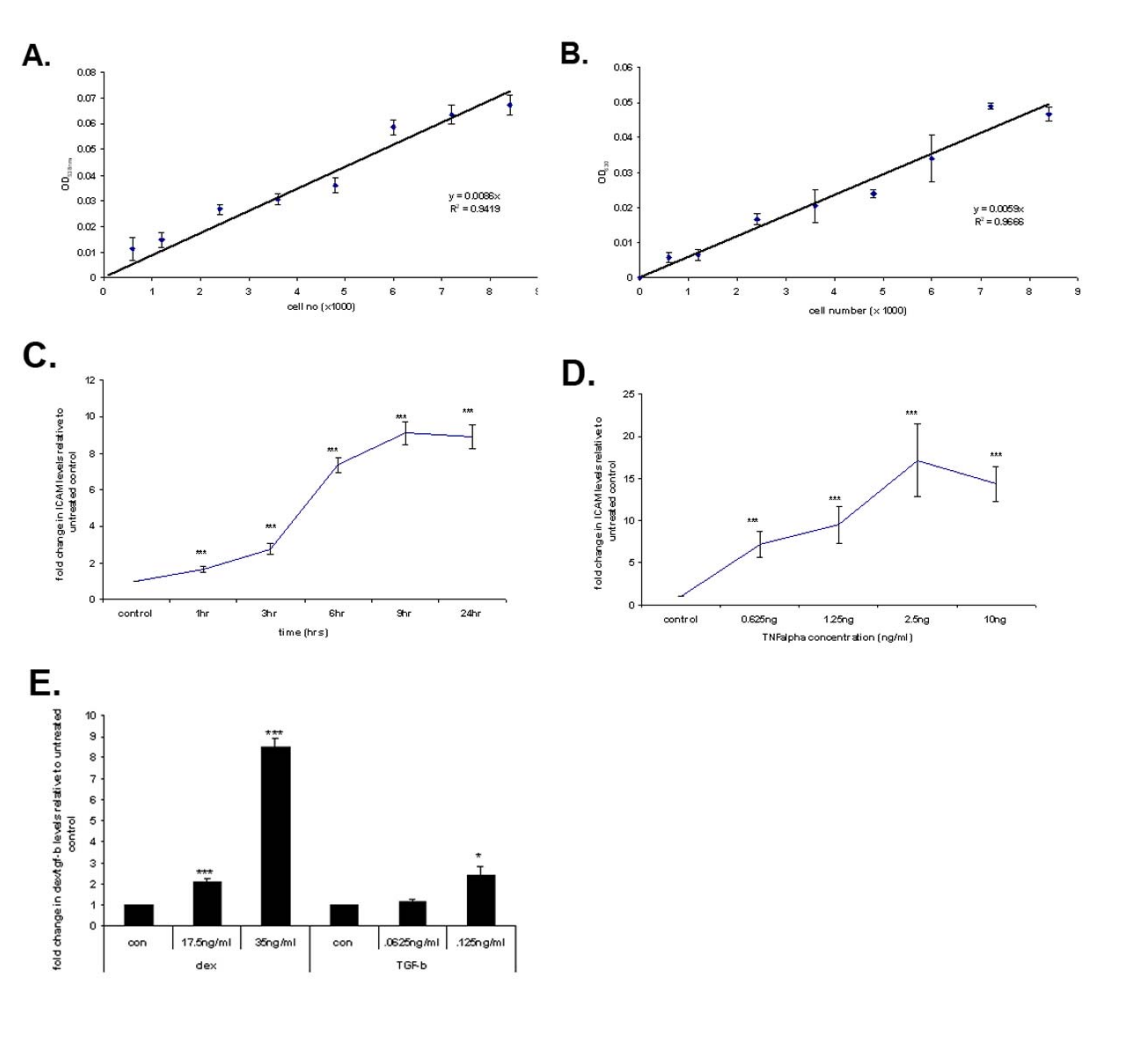
**Validation of whole cell elisa protocol for ICAM, and CTGF**. Fig. S1 A, B; standard curves for cell # vs. OD for ponceau red (protein) and methylene green (DNA) respectively. Fig. S1 C, D; time and dose response curves for ICAM detected by whole cell elisa vs. TNFα. Fig. S1E; CTGF levels as measured by whole cell ELISA vs. DEX and TGF-β concentration. Fig. S1A, B; data is presented as zeroed OD ± SEM. Fig. S1. C, D, E, F; data is presented as fold change relative to untreated control ± SEM. All data is the mean of several independent experiments. (Significance determined at; *p ≤ 0.05, **p ≤ 0.01, ***p ≤ 0.005.)

For differentiation experiments, as increased production of proteinaceous extracellular matrix made cell number determination using protein staining inaccurate, DNA staining with 1% methylene green (dH_2_O) was used. Cells were stained/destained in a similar manner to ponceau red, and dye extracted was read at 600 nm, with cell number per well determined using an appropriate standard curve. (standard curves for both ponceau red and methylene green are shown in Fig. 1a and 1b).

### Whole Cell ELISA

MSCs were cultured in 96 well plates and treated as described above. Following treatment cells were washed with PBS and fixed with 100 ul ice cold methanol per well. Cell number was determined as described above.

Following determination of cell number the cells were, soulbilised with 1% tritonX/PBS for 5 mins, and blocked in 100 ul PBS containing 10% BSA for 30 mins. The cells were then treated with 100 ul of appropriate primary antibody dilution [rabbit anti-CTGF (santa cruz): 1/200, mouse anti-ICAM 1/200 (santa cruz); all dilutions in PBS/10% BSA] and incubated for one hour at room temperature. Following incubation wells were washed three times with PBS containing 0.05% tween, and incubated with appropriate concentrations of horse radish peroxidase (HRP) labeled secondary antibodies [anti-rabbit HRP (sigma): 1/300, anti-mouse HRP (sigma): 1/250; all dilutions in PBS/10%BSA]. Wells incubated with secondary antibody were included for each treatment as a control for non-specific binding and to act as blanks.

Following incubation with secondary antibody, the wells were washed four times PBS containing 0.05% tween, and allowed to air dry. SigmaFAST OPD was prepared as per the manufacturers instructions, and 100 ul of the solution added to all wells. The plate was then incubated at room temperature in the dark for 15 mins. After incubation the reaction was stopped by the addition of 50 ul 0.1 M acetic acid, and the plates read on a spectrometer at 450 nm, correction wavelength 600 nm. OD values for each well were zeroed by subtraction of the value of the appropriate secondary-only control, and the zeroed value normalized to cell number.

The optimum concentraction of primary/secondary antibodies were determined using concentration gradients. The assay was also validated for each ICAM, and CTGF antibodies using an appropriate positive control treatment, TNFα – ICAM [26], and dexamethasone/TGFβ – CTGF [27,28] (Fig. 1c, d, e, respectively).

### BMP-2 ELISA

Post treatment cell supernatants were collected, centrifuged at 1000 rpm/5 min to remove cellular debris, aliquoted, and stored at -80°C. A commercially available ELISA kit was then used to determine the concentration of BMP-2 (R&D systems, USA). The assay was carried out and analysed as per manufacturer's instructions.

### Transcription Factor Activity Assays

RUNX-2 and PPARγ transcription factor activity was determined using a commercial TransAM™ assay as per manufacturers instructions (Active Motif, Belgium). TransAM Kits are highly sensitive ELISA based assays that enable the detection of activated transcription factors in mammalian tissue and cell culture extracts. TransAM Kits are easy-to-use and up to 100-fold more sensitive than gel-shift assays. The kits comprise a 96 well plate on which a consensus sequence for the transcription factor in question has been imobilised (RUNX-2, 5'-AACCACA-3'; PPARγ, 5'-AACTAGGTCAAAGGTCA-3' [PPRE]). Nuclear extracts are then added to the plates and allowed to bind. Positive controls were also included, namely nuclear extracts from cell lines tranfected with constructs over-expressing RUNX-2/PPARγ. Wild type and mutated consensus sequence could also be included to monitor the specificity of the assay. Protein bound to the consensus sequence was then treated with specific primary antibody, which was then be detected using HRP labeled secondary antibody and a colourimetric substrate. The assay was read at 450 nm, and readings were normalized to protein concentration.

### Statistical Analysis

Statistical analysis was performed using students unpaired t-test, with significance determined at *p ≤ 0.05, **p ≤ 0.01, and ***p ≤ 0.005 relative to untreated control, and ^p ≤ 0.05, ^^p ≤ 0.01, and ^^^p ≤ 0.00 relative to differentiated control.

## Results

### Treatment with HIV proteins p55-gag and REV dysregulates MSC osteogenic differentiation

**Figure 2:**
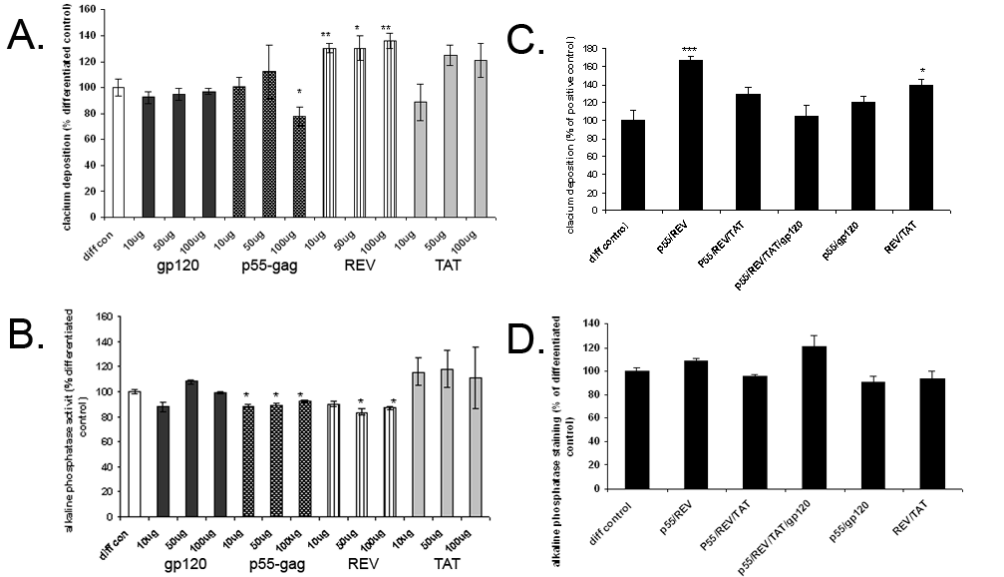
**Effect of varying concentrations and combnations of HIV1 proteins on MSC osteogenesis**. MSCs were cultured in a 96-well plate format and induced to differentiate in the presence and absence of 10–100 ng/ml of HIV REV, gp120, p55-gag, and TAT. Untreated, differentiated cells were included as controls. Cells were harvested at 15 days post induction of differentiation, fixed and stained for either calcium deposition (alizarin red) or alkaline phosphatase (BCIP/NBT). Plates were first photographed, then the stain extracted, and the degree of staining quantified spectrophotometrically and normalised to cell number. Fig 2A; calcium staining normalised to cell number for diferentiating MSCs (diff con), and differentiating MSCs exposed to 10–100 ng/ml HIV gp120, p55-gag, REV and TAT proteins, data expressed as percentage of untreated control ± SEM. Fig 2B; ALP staining normalised to cell number, for diferentiating MSCs (diff con), and differentiating MSCs exposed to 10–100 ng/ml HIV gp120, p55-gag, REV and TAT. proteins, data expressed as percentage of untreated control ± SEM. Differentiating MSCs were also incubated with various combinations of HIV1 proteins (all proteins at 100 ng/ml), and there affects on calcium deposition and alkaline phosphatase staining assayed and quantified as described. Fig 2C; Fig 2A; calcium staining normalised to cell number for differentiating MSCs (diff control), and differentiating MSCs exposed to either 100 ng/ml REV/p55, 100 ng/ml p55/REV/TAT, 100 ng/ml p55/REV/TAT/gp120, 100 ng/ml p55/gp120, or 100 ng/ml REV/TAT proteins, data expressed as percentage of untreated control ± SEM. Fig 2D; ALP staining normalised to cell number, for differentiating MSCs (diff con), and differentiating MSCs exposed to either 100 ng/ml REV/p55, 100 ng/ml p55/REV/TAT, 100 ng/ml p55/REV/TAT/gp120, 100 ng/ml p55/gp120, or 100 ng/ml REV/TAT proteins, data expressed as percentage of untreated control ± SEM. All data is the mean of several independent experiments. (Significance determined at; *p ≤ 0.05, **p ≤ 0.01, ***p ≤ 0.005 relative to undifferentiated control, ^p ≤ 0.05, ^^p ≤ 0.01, ^^^p ≤ 0.005 relative to differentiated control).

Cells were cultured in ostegenic conditions in a 96-well plate format for 15 days in the presence of varying concentrations (10 ng/ml, 50 ng/ml, 100 ng/ml) of HIV-1 proteins gp120, p55-gag REV and TAT. Untreated, differentiated cells were included as controls. After 15 days the cells were harvested, fixed and stained for calcium deposition (alizarin red) (Fig. 2A) and alkaline phosphatase activity (BCIP/NIB) (Fig. 2B), known markers of osteogenic differentiation. Stained cells were photographed and the dyes were extracted and quantified spectrophotometrically. Only p55-gag and REV were seen to significantly alter either alkaline phosphatase activity and calcium deposition relative to differentiated controls; all concentrations of REV significantly increased calcium deposition (> 30% relative to differentiated control, p ≤ 0.05), while the higher concentrations were also seen to alter alkaline phosphatase activity. All concentrations of p55-gag were seen to significantly reduce alkaline phosphatase activity (> 10% relative to differentiated control, p ≤ 0.05), while only treatment with 100 ng/ml p55-gag was seen to reduce calcium deposition significantly (22.4 ± 7.1% relative to differentiated control, p ≤ 0.05). TAT proteins also induced increases in both calcium deposition and alkaline phosphatase activity at most of the concentrations examined, however. The effect of combinations of the proteins (all used at 100 ng/ml) on MSC osteogenesis was also examined; combination of p55-gag or TAT with REV did not attenuate the increase in calcium deposition observed with REV alone (Fig. 2C), while none of the other combinations of proteins significantly altered either alkaline phosphatase or calcium deposition relative to the differentiated control (Fig. 2C, D).

**Figure 3:**
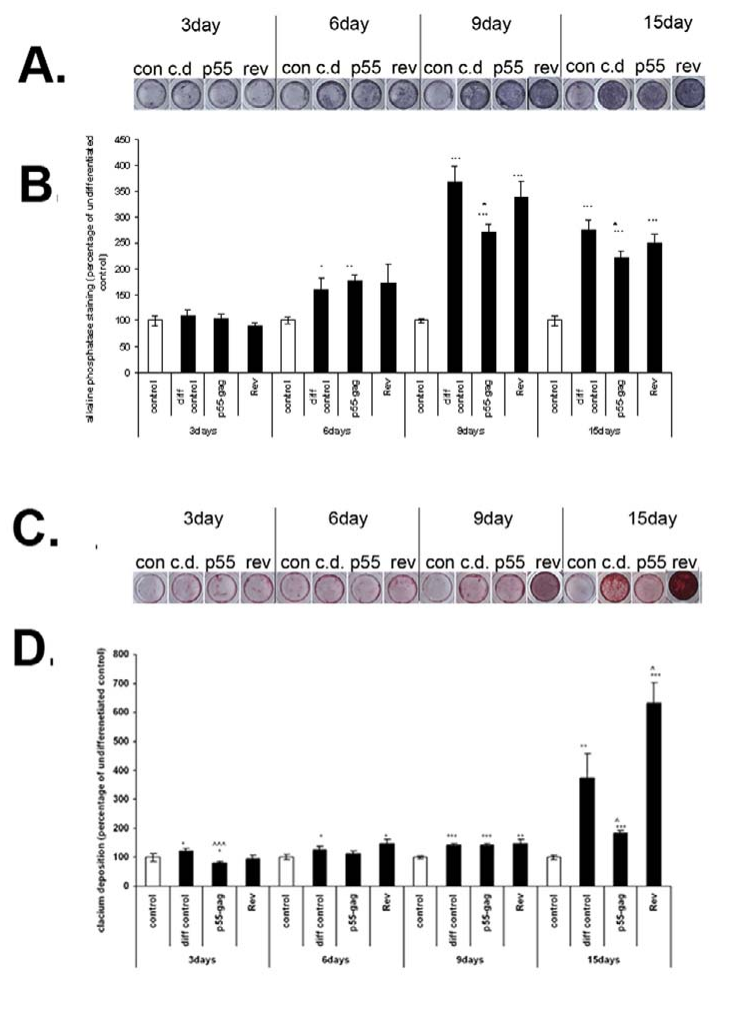
**Effect of HIV1 p55-gag and REV on MSC osteogenesis**. MSCs were cultured in a 96-well plate format and induced to differentiate in the presence and absence of HIV REV and p55-gag. Untreated, undifferentiated cells were included as controls. Cells were harvested at 3, 6, 9 and 15 days post induction of differentiation, fixed and stained for either calcium deposition (alizarin red) or alkaline phosphatase (BCIP/NBT). Plates were first photographed, then the stain extracted, and the degree of staining quantified spectrophotometrically and normalised to cell number. Fig 3a; alkaline phosphatase (ALP) staining in untreated MSCs (con), diferentiating MSCs (cd), and differentiating MSCs exposed to HIV p55-gag and REV proteins. Fig 3b; ALP staining normalised to cell number, data expressed as percentage of untreated control ± SEM. Fig 3c; calcium staining in untreated MSCs (con), diferentiating MSCs (cd), and differentiating MSCs exposed to HIV p55-gag and REV proteins. Fig 3d; calcium staining normalised to cell number, data expressed as percentage of untreated control ± SEM. All data is the mean of several independent experiments. (Significance determined at; *p ≤ 0.05, **p ≤ 0.01, ***p ≤ 0.005 relative to undifferentiated control, ^p ≤ 0.05, ^^p ≤ 0.01, ^^^p ≤ 0.005 relative to differentiated control).

To more closely examine the individual impact of the REV and p55-gag proteins on the osteogenic process, MSCs were cultured in osteogenic media for 15 days in the presence of either100 ng/ml p55-gag or REV, and time points taken at 3, 6, 9 and 15 days. Again cells were fixed, and stained for ALP activity (Fig. 3A, B) and calcium deposition (Fig. 3C, D).

ALP showed maximum induction in the differentiated control cells at 9 days, with an increase of 167 ± 32% (p ≤ 0.001) relative to undifferentiated control. The degree of increase in ALP staining (70 ± 18.9%, p ≤, n = 6) in cells treated with p55-gag was significantly lower than the differentiated control (p ≤ 0.05, n = 6), while in cells treated with REV there was no significant difference in the degree of induction. A similar pattern was observed at 15 days, albeit with a lower overall level of ALP induction in the differentiated control (74 ± 18.9% relative to untreated control, p ≤ 0.001, n = 6).

The maximum increase in calcium deposition was observed at 15 days; with an increase of 173 ± 73% (p ≤ 0.05, n = 6) in untreated, differentiated cells. Treatment with p55-gag significantly reduced the degree of calcium deposition relative to differentiated control, while REV increased it (p ≤ 0.05 relative to differentiated control, n = 6); with increases of 82.4% ± 10% and 430 ± 71% relative to untreated control respectively (p ≤ 0.001, n = 6).

### HIV proteins induce changes in calcium deposition, alkaline phosphatase activity, and lipid levels in non-differentiating MSCs

Having demonstrated that the HIV proteins p55-gag and REV could disregulate the osteogenic differentiation process in MSCs, we attempted to determine if these proteins, in and of themselves, could induce any degree of differentiation.

**Figure 4:**
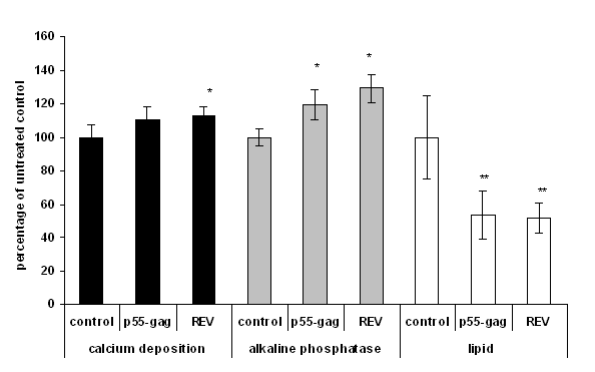
**Effect of hiv proteins on non differentiating MSCs**. MSCs were cultured in 96-well plates in the presence of 100 ng/ml HIV p55-gag and REV for 7 days. Untreated cells were included as controls. Cells were harvested and the stained for alkaline phosphatase, calcium deposition, and lipid accumulation (Fig. 4). Following staining dyes were extracted, the degree of staining quantified spectrophotometrically and normalised to cell number. Data expressed percentage of untreated control ± SEM. All data is the mean of several independent experiments. (Significance determined at; *p ≤ 0.05, **p ≤ 0.01, ***p ≤ 0.005)

Cells were cultured for 7 days in the presence of 100 ng/ml p55-gag and REV. Untreated cells were included as controls. Cells were harvested, fixed, and stained for calcium deposition (alizarin red), for ALP activity (BCIP/NIB), and lipid levels (oil red-o) (Fig. 4). Stained cells were photographed and the dye was extracted and quantified spectrophotometrically.

REV increased calcium deposition slightly (12.2 ± 5.4%, p ≤ 0.05, n = 6), while both REV and p55-gag induced increases in alkaline phosphatase staining (29.1 ± 8.4% and 19.3 ± 8.9% respectively, p ≤ 0.05, n = 6). Both proteins significantly reduced the levels of lipid staining, with p55-gag induced a decrease of 46.6 ± 14% and REV and decrease of 48.4 ± 8.6% respectively (p ≤ 0.01, n = 6).

### P55-gag and REV alter BMP-2 secretion, cellular levels of CTGF, and RUNX-2 activity in differentiating MSCs

Having determined that p55-gag and REV could disregulate the osteogenic process in MSCs, we examined the effect of treatment with HIV proteins on the levels and activity of three molecules important in the process of osteogenesis in MSCs, (BMP-2, CTGF and RUNX-2), over the course of differentiation.

**Figure 5:**
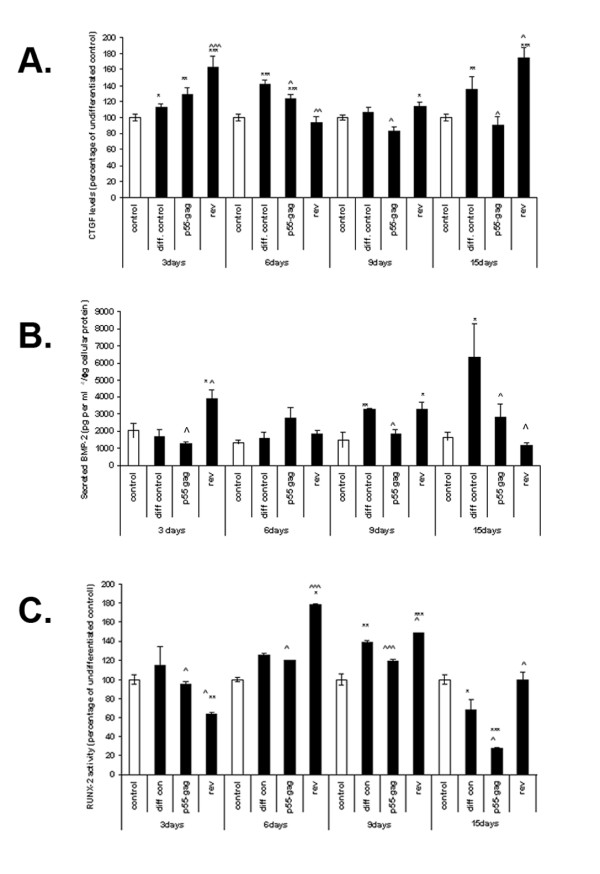
**HIV1 proteins alter RUNX-2 activity, BMP-2 secretion and cellular levels of CTGF in differentiating MSCs**. MSCs were cultured in a 96-well plate format and induced to differentiate in the presence and absence of 100 ng/ml of HIV REV and p55-gag. Untreated, undifferentiated cells were included as controls. Cells were harvested at 3, 6, 9 and 15 days post induction of differentiation; whole cell ELISA was performed for CTGF (Fig. 5A), media fractions were analysed for BMP-2 levels (Fig. 5B), while nuclear extracts were assessed for RUNX-2 activity (Fig. 5C), Data expressed percentage of untreated control ± SEM (A, C,), or pg BMP-2 per ml^-1^/μg cellular protein extracted (B) ± SEM. All data is the mean of several independent experiments. (Significance determined at; *p ≤ 0.05, **p ≤ 0.01, ***p ≤ 0.005 relative to undifferentiated control, ^p ≤ 0.05, ^^p ≤ 0.01, ^^^p ≤ 0.005 relative to differentiated control).

Cells were cultured in osteogenic media for 15 days in the presence of 100 ng/ml p55-gag and REV. Both untreated and undifferentiated cells were included as controls. Cells were harvested at 3, 6, 9 and 15 days; cells were fixed and cellular levels of CTGF determined using whole cell elisa (Fig. 5A), media fractions were harvested and analysed for BMP-2 levels (Fig. 5B), while nuclear extracts also were prepared and analysed for RUNX-2 activity (Fig 5C).

CTGF levels (Fig. 5A) in the differentiated control show a significant increase relative to undifferentiated control at day 3 and day 6 (13.2 ± .3% and 41.7 ± 5% respectively; p ≤ 0.001, n = 6). In contrast, levels in cells treated with REV were significantly higher than differentiated control in day 3, and 15, and lower in day 6 (p ≤ 0.05 relative to differentiated control, n = 6). REV also induces a small but significant increase relative to undifferentiated control at day 9 (13.7 ± .47%, p ≤ 0.05). Levels in cells treated with p55-gag are significantly lower than the differentiated control at day 6, while there is a small but significant decrease relative to undifferentiated control at day 9 (16.8 ± 5%, p ≤ 0.05, n = 6).

Levels of BMP-2 (Fig. 5B) secretion were altered in differentiated controls at days 9 and 15 with increases of 125.1 ± 2.1% and 282.7 ± 30% relative to undifferentiated control respectively (p ≤ 0.05, n = 4). In contrast the level of BMP-2 secretion in cells treated with REV increased at day 3, (91.2 ± 14.2% relative to undifferentiated control, p ≤ 0.05, n = 4), which was also significantly greater than the differentiated control (p ≤ 0.05), and decreased at day 15 (28 ± 9.6% relative to undifferentiated control) which was also significantly lower than the differentiated control (p ≤ 0.05, n = 4). Levels of BMP-2 secretion in cells treated with p55-gag were significantly lower than undifferentiated control at day 3 (p ≤ 0.05, n = 3), and significantly lower than levels of the differentiated control at days 9 and 15 (p ≤ 0.01 and p ≤ 0.05 respectively, n = 4).

The levels of RUNX-2 activity (Fig. 5C) in untreated differentiating cells did not alter significantly relative to undifferentiated control until day 9, when a significant increase of 39 ± 9% is observed (p ≤ 0.05, n = 3). By day 15 the levels of RUNX-2 in differentiating cells activity dropped significantly relative to undifferentiated control by 32 ± 11% (p ≤ 0.05, n = 3). Differentiating cells treated with REV showed earlier and significantly greater increases in activation (day 6; 78 ± 0.4%. day 9; 48 ± 0.8%: n = 3, p ≤ 0.05 relative to undifferentiated control), and did not exhibit the drop in activity at day 15 shown by the differentiated control. However levels at day 3 are significantly lower than those of both the control and differentiated control cells. Treatment with p55-gag significantly reduces levels of RUNX-2 activity relative to differentiated control at all time points.

### HIV proteins induce changes in levels of RUNX-2 and PPARγ activity in non-differentiating MSCs

Having shown that both p55-gag and REV can induce changes in the levels of osteogenic and adipogenic makers in non-differentiating MSCs, we examined their effect on the transcription factors PPARγ and RUNX-2, which are know to drive an adipogenic or osteogenic phenotype respectively.

**Figure 6:**
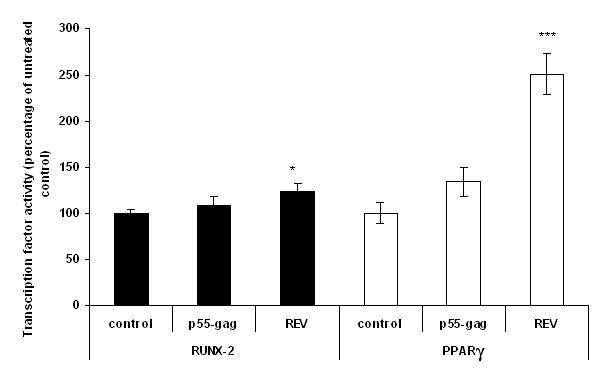
**Effect of HIV1 proteins on transcription factor activity in non differentiating MSCs**. MSCs were cultured in the presence of 100 ng/ml HIV p55-gag and REV for 7 days. Untreated cells were included as controls. Cells were harvested and nuclear extracts were prepared and analysed for RUNX-2 and PPARγ activity. Data expressed as percentage of untreated control ± SEM. All data is the mean of several independent experiments. (Significance determined at; *p ≤ 0.05, **p ≤ 0.01, ***p ≤ 0.005.)

Cells were treated with 100 ng/ml p55-gag and REV for 7 days. Untreated cells were included as controls. Nuclear extracts were prepared and analysed for RUNX-2 and PPARγ activity (Fig. 6).

REV induced an increase in the activity of RUNX-2 of 23.3 ± 8% (p ≤ 0.05, n = 4). In addition REV also induced a significant increase in PPARγ activity (151 ± 21.8%, p ≤ 0.005, n = 4) (Fig. 6A). Although p55-gag induced smaller increases in the levels of activity of both proteins, these increases were not significant.

## Discussion

Previous studies by our groups have indicated that the HIV1 gp120 and p55-gag proteins could regulate osteoblast function. In this study we further examined the effect of exposure to HIV proteins on MSC ostegenic differentiation over a 15 day time course. Initial experiments examined the effects of an array of HIV-1 proteins; the capsid protein p55-gag, the coat protein gp120, and the proteins transcriptional regulatory proteins REV and TAT. These experiments demonstrated that REV and p55-gag, alone and in combination, could significantly alter MSC osteogenesis. To further determine the effect of REV and p55-gag on the key signalling events involved in osteogeneis, we examined the functional markers of ALP activity and calcium deposition, and also examined RUNX-2 activity, BMP-2 secretion, and total cellular levels of CTGF.

The generally accepted model of the progression of osteogenesis is as follows, there is an early proliferative stage (day 0–4) followed by a period of matrix maturation day (3–14), and finally the mineralization stage (day 13–15), indicating the transition of the pluripotent MSC to a committed osteoprogenitor, preosteoblast, early osteoblast and finally a mature osteoblast [23,23]. We examined a number of important signalling proteins and osteoblastic markers over the course of differentiation (ALP, calcium deposition, BMP-2, RUNX-2, CTGF). Our findings in un HIV treated differentiating cells, correlate with the accepted model of osteogenic differentiation; in our model (see Fig. 6), an increase in CTGF production at day 3–6 was consistent with the onset of matrix maturation in the preosteoblast, while increases in BMP-2 secretion, RUNX-2 activity and ALP activity between day 6–9 was indicative of the preosteoblast stage, with the final increase in mineralization observed at day 15 as the cell became a mature osteoblast. Interestingly, we demonstrated a decrease in RUNX-2 activity at day 15, an observation which is in keeping with findings that suggest that although RUNX-2 expression is essential for osteoblast development and function, in the maturing cell type its overexpression blocks terminal differentiation and increases bone resorption [29].

**Figure 7:**
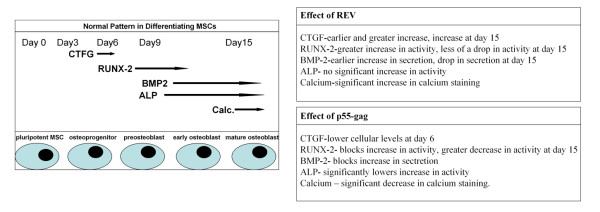
Summary of findings for differentiation experiments.

Treatment with both p55-gag and REV altered both the final degree of differentiation and the levels of many of the parameters examined over the 15 day timecourse. P55-gag significantly reduced the degree of osteogenesis (as measured by ALP levels and calcium staining) at day 15. Reductions in RUNX-2 activity relative to differentiated control was also observed at all time points, and the secretion of BMP-2 is also reduced relative to differentiated control at 9 and 15 days. In addition, the levels of CTGF in p55-gag treated cells was lower at day 6 than those in untreated differentiating cell. Taken together these results suggest that p55-gag interferes with the osteogenic function by inducing changes in the levels of key signalling molecules. Previous studies by our group showed that p55-gag could impair the function of mature osteoblasts, and alter the levels of RUNX-2, and BMP-2 to a similar degree [24]. Our findings with regard to differentiating MSCs clearly build on this (Fig. 7).

In contrast to the anti-osteogenic role played by p55-gag, REV clearly has a pro-osteogenic effect. Not only was the magnitude of several of the examined parameters (such as calcium deposition and RUNX-2 activity) greater over many of the time-points examined, the osteogenic process seems to be accelerated, with earlier increases in the levels of CTGF and BMP-2 observed at day 3. The levels of RUNX-2 and CTGF were also higher at day 15 than those of untreated, differentiated cells; this may be indicative of further osteogenesis occurring, or the beginning of a process of differentiation into another cell type such as chondrocytes (Fig. 7) [22,30].

Having demonstrated that HIV proteins interfere with the normal process of MSC osteogenic differentiation; we studied the possibility that HIV proteins alone could induce some degree of differentiation. We treated MSCs with p55-gag and REV for 7 days (an intermediate time point at which time we proposed any significant molecular/functional shift towards an osteogenic/adipogenic phenotype would be evident) and examined the functional markers of osteoblastogenesis (ALP and calcium deposition), and adipogenesis (lipid accumulation). We also examined the activity levels of the key transcription factors RUNX-2 and PPARγ. At a functional level both proteins (and REV in particular) were seen to increase osteogenic markers, and decrease lipid staining; findings which would seem to indicate that they are driving a pro-osteogenic phenotype. However, at the transcription factor level, although REV significantly increased RUNX-2 activity, it also increased the levels of the pro-adipogenic PPARγ (as did p55-gag, although it was not significant [p = 0.097]). As the actions of PPARγ and RUNX-2 in MSC differentiation are generally considered to oppose each other [17], with, for example, suppression of PPARγ being necessary for osteogenesis to occur [31], the results of these experiments suggest that although the proteins may disrupt MSC function and signalling to a certain degree, it seems unlikely that they in and of themselves are capable of inducing differentiation. It may be interesting to ascertain if HIV proteins alter other MSC functions, such as proliferation and migration. However we feel such experiments are beyond the scope of the current study.

The findings of our study suggests that HIV proteins may regulate the production of osteoblasts from mesenchymal progenitors, through interfering with the process of osteogenesis. In vivo, this alteration in functional osteoblast numbers may drive decreased bone mineral density seen in HIV patients. Previous studies have demonstrated that exposure of MSC to HIV-1 virus *in vitro *can impair the clonogenic potential of MSCs in a TAT protein dependent manner [32], and our findings fit into a general hypothesis of a HIV mediated disregulation of MSC function. The exact contribution of virus and treatment to the process of loss of BMD observed in HIV1 infected patients remains unclear. Although successful HAART can reduce serum levels of virus to almost undetectable levels, residual latent infection in various tissue types can remain [33]. It is also plausible that exposure to HIV1 proteins early in infection or infection of MSC/OB cell types 'prime' these cells to the inhibitory effects of HAART. Furthermore, MSC/OB cell types interact with numerous other cell types throughout their development, and the disruption of these interactions, rather than disruption of MSC/OB themselves, may be a contributory factor in the clinically observed losses in bone mass. The possibility that both MSCs and hematopoietic cell types can be affected by secreted inhibitory factors from HIV1 infected stroma has been considered, and it has been demonstrated in long term tissue culture models that that altered support of hematopoietic stem cells occurred only when the stromal layer was susceptible to infection with HIV1 [33,34]. It is worth noting that altough the treatment of cells with exogenously added HIV proteins is a useful model it is not ideal. In the future either experiments involving a combination of intercellular expression of certain proteins with extracellular treatment of others, or attempting to infect cultured MSCs with live HIV virus (possibly using methodologies similar to that used by Wang *et al*. [32]), are likely areas of future study as we attempt to extend this hypothesis.

## Conclusion

In conclusion, our findings suggest that HIV p55-gag and REV alter the degree of osteogenesis in differentiating MSCs by perturbing the timing and magnitude of important osteogenic signals and processes. These data lend further weight to the hypothesis that HIV infection drives alterations in bone phenotype and provide a potential mechanism for the evolution of this complication of HIV1 infection.

## Competing interests

The author(s) declare that they have no competing interests.

## Authors' contributions

EJC was involved with development of the concept, acquisition of the majority of the data, analysis of data and the drafting of paper. HI acquisition of data for the assay validation experiments and analysis of the data.WGP contributed to the development of concept, and approval of document for final submission. PPD contributed to the development of concept and the revision of paper. All authors read and approved final accepted document.

## Pre-publication history

The pre-publication history for this paper can be accessed here:


